# Distal airway epithelial progenitor cells are radiosensitive to High-LET radiation

**DOI:** 10.1038/srep33455

**Published:** 2016-09-23

**Authors:** Alicia M. McConnell, Bindu Konda, David G. Kirsch, Barry R. Stripp

**Affiliations:** 1 Department of Cell Biology, Duke University Medical Center, Durham, NC, 27708, USA; 2 Lung and Regenerative Medicine Institutes, Department of Medicine, Cedars-Sinai Medical Center, Los Angeles, CA, 90048, USA; 3 Departments of Radiation Oncology and Pharmacology & Cancer Biology, Duke University Medical Center, Durham, NC, 27708, USA

## Abstract

Exposure to high-linear energy transfer (LET) radiation occurs in a variety of situations, including charged particle radiotherapy, radiological accidents, and space travel. However, the extent of normal tissue injury in the lungs following high-LET radiation exposure is unknown. Here we show that exposure to high-LET radiation led to a prolonged loss of *in vitro* colony forming ability by airway epithelial progenitor cells. Furthermore, exposure to high-LET radiation induced clonal expansion of a subset of progenitor cells in the distal airway epithelium. Clonal expansion following high-LET radiation exposure was correlated with elevated progenitor cell apoptosis, persistent γ-H2AX foci, and defects in mitotic progression of distal airway progenitors. We discovered that the effects of high-LET radiation exposure on progenitor cells occur in a p53-dependent manner. These data show that high-LET radiation depletes the distal airway progenitor pool by inducing cell death and loss of progenitor function, leading to clonal expansion. Importantly, high-LET radiation induces greater long-term damage to normal lung tissue than the relative equivalent dose of low-LET γ-rays, which has implications in therapeutic development and risk assessment.

Humans are exposed to radiation during circumstances such as medical diagnostic or therapeutic treatment, high altitude or space travel, and radiological warfare or accidents. In each of these situations, the extent of damage to normal tissues varies according to radiation dose and quality. Radiation quality can be categorized according to linear energy transfer (LET), or the amount of energy deposited as a particle traverses the tissue^1^. X-rays and γ-rays, types of low-LET radiation, deposit energy in a diffuse manner, whereas heavy ions, types of high-LET radiation, deposit energy along more concentrated tracks^2^. Due to the differences in energy deposition and subsequent DNA damage, heavy ions, such as ^12^C, are increasingly being utilized in radiation therapy treatment^1^,^3^. Additionally, during space travel, astronauts can be exposed to galactic cosmic rays, which contain high charge and energy (HZE) ions including ^56^Fe and ^28^Si^4^. A greater understanding of the extent of damage inflicted on normal tissue following high-LET radiation exposure and how this compares to low-LET radiation exposure is important for further development of heavy ions for therapy and for risk assessment of normal tissue damage during radiotherapy or deep space travel.

The carcinogenic effects of ionizing radiation exposure are well established^4^,^5^. Recent literature has shown that tumors frequently arise from resident tissue progenitor cells^6^,^7^. However, the relationship between progenitor cell injury by radiation and cancer development is unknown. Progenitor cell sensitivity and response to radiation exposure has been studied in a number of organs, including the epidermis, mammary gland, intestine, and hematopoietic system, and is largely tissue-specific^8^,^9^,^10^,^11^,^12^,^13^. Yet, less is known about the effects of radiation on progenitor cells in the pulmonary system. Club cells, previously known as Clara cells, specifically express the protein *Scgb1a1* and function as regional progenitors that maintain the distal conducting airway of the murine lung^14^. We previously reported that immediately following whole-body radiation exposure, these club progenitor cells exhibit a dose-dependent decrease in *in vitro* colony forming ability, but that a subset of these cells undergo radiation-induced clonal expansion without an increase in the overall rate of epithelial cell proliferation *in vivo*^15^. However, in this study we were unable to resolve differences arising from radiation quality in low- and high-LET radiation. Additionally, we did not identify a mechanism by which radiation depletes the pool of progenitor cells in the lung and leads to clonal expansion.

Here we show that radiation exposure injures airway epithelial club progenitor cells in a region-specific and quality-dependent manner. High- but not low-LET exposure resulted in the persistent loss of colony-forming progenitors. Using a novel whole mount imaging and quantification technique, we reveal that high-LET radiation exposure causes substantial progenitor cell expansion in the distal, but not proximal airways. This expansion is induced by apoptosis, senescence, and defects in mitotic progression among neighboring progenitor cells that are regulated in a p53-dependent manner. Together, these data indicate that high-LET, but not low-LET radiation leads to prolonged impairment of distal airway epithelial progenitor cells, leaving fewer progenitors to maintain the airway during homeostasis.

## Results

### Airway progenitors exposed to high-LET radiation have a prolonged decrease in colony forming ability

**Figure 1:**
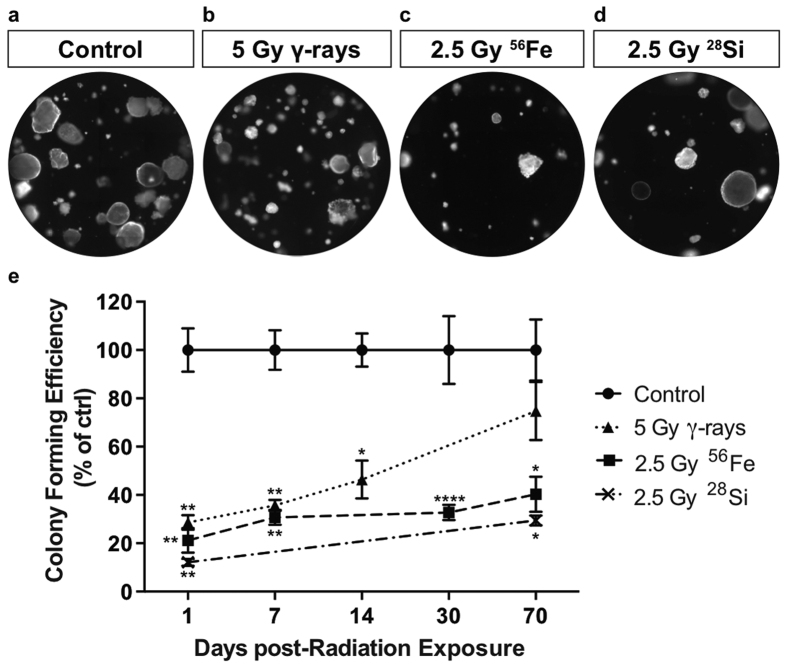
Epithelial progenitor cells of mice exposed to low- but not high-LET radiation recover colony forming ability over time. Mice were exposed to 2.5, or 5 Gy high- or low-LET radiation respectively and epithelial cells were isolated at 1, 7, 14, 30, or 70 days post-radiation exposure. (**a**–**d**) Fluorescent images of 3D colonies grown from RFP+ epithelial cells of mice that were irradiated 70 days prior to isolation. (**e**) Colony forming efficiency of epithelial cells isolated from mice at various time points following radiation exposure. Colony forming efficiency is expressed as a percent of the unirradiated control. Significance of differences relative to unirradiated control is indicated by: *p < 0.05, **p < 0.01, ***p < 0.001, ****p < 0.0001.

To determine the long-term effect of radiation exposure on club progenitor cells, we used an *in vitro* colony-forming assay to measure their clonogenic capacity. We previously used full dose response curves of low-LET X-rays and high-LET 600 MeV/nucleon ^56^Fe to assess the relative biological effectiveness (RBE) with an *in vitro* colony-forming assay as the endpoint, which revealed an RBE of approximately 2^15^. In order to achieve a similar level of injury and loss of progenitor cell function, mice that ubiquitously expressed a membrane localized RFP were irradiated with 5 Gy low-LET γ-rays, 2.5 Gy 600 MeV/nucleon ^56^Fe, or 2.5 Gy 300 MeV/nucleon ^28^Si. Fluorescent lung epithelial cells were isolated at various times post-radiation exposure and were plated in a 3D co-culture system containing unirradiated non-fluorescent fibroblasts. As expected based on previous studies^15^, whole-body exposure to isotropic doses of ionizing radiation led to an acute loss of progenitor cell colony-forming ability at 1 day post-exposure regardless of radiation quality. However, the magnitude of this decline differed slightly between radiation qualities. Exposure to 2.5 Gy ^28^Si resulted in the greatest decrease in colony-forming ability (15% of control), followed by 2.5 Gy ^56^Fe (20% of control), with 5 Gy γ-rays showing the least reduction (25% of control) (Fig. 1e and Supplemental Fig. 1). After exposure to 5 Gy γ-rays the initial decline in epithelial colony-forming ability recovered and was not different from that of un-irradiated controls when isolated 70 days after exposure (Fig. 1a,b,e). However, the colony forming ability of progenitors from mice exposed to high-LET radiation, either ^56^Fe or ^28^Si, remained significantly decreased compared to control at the 70 day recovery time point (Fig. 1c–e). Taken together, these results suggest that exposure to high-, but not low-LET radiation leads to prolonged defects in the ability of club progenitor cells to proliferate and contribute to maintenance of the airway epithelium.

### Low-LET radiation induces moderate airway progenitor expansion *in vivo*

**Figure 2:**
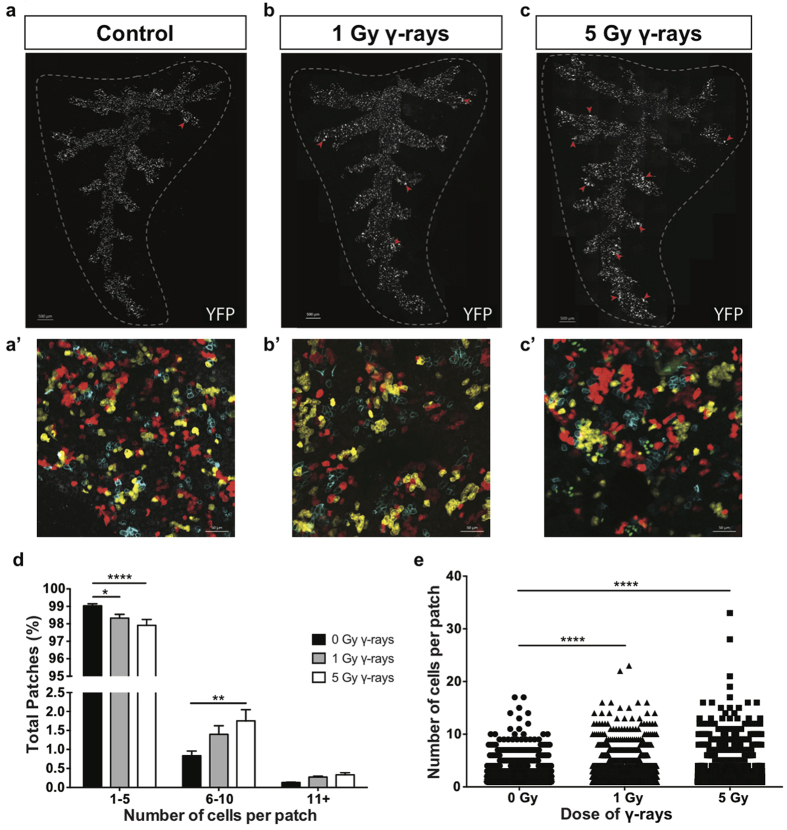
Low-LET radiation induces moderate expansion of subsets of lineage-labeled airway progenitor cells. *Scgb1a1-CreER; Rosa26R-Confetti* mice were exposed to 0, 1, or 5 Gy γ-rays and lungs were harvested after 70 days. (**a**–**c**) Tiled image of the native YFP fluorescence (white) in a whole mount, microdissected, Sca*l*e treated accessory lobe 70 days post-γ radiation exposure. Red arrows indicate medium or large patches. Scale bar represents 500 μm. (**a’**–**c’**) Whole mount 4-color fluorescence images of native confetti fluorescence (cytoplasmic RFP, nuclear GFP, cytoplasmic YFP, and membrane CFP) 70 days post-γ radiation exposure corresponding to the tiled image above. Scale bar represents 50 μm. (**d**) Relative frequency of YFP patches containing various numbers of cells 70 days post-γ-ray exposure. (**e**) Number of YFP cells per patch 70 days post-γ-ray exposure. Significance of differences between groups is indicated by: *p < 0.05, **p < 0.01, ****p < 0.0001.

Given our findings that radiation induces quality-dependent differences in club progenitor cell behavior *in vitro*, we hypothesized that quality-dependent differences in progenitor cell dynamics existed *in vivo* as well. To assess progenitor cell dynamics, we developed a sensitive whole mount imaging system to detect clonal expansion. *Scgb1a1-CreER; Rosa26R-Confetti* mice were exposed to tamoxifen one week before radiation exposure. This induced recombination and stable expression of one of four fluorescent proteins (cytoplasmic RFP, nuclear GFP, cytoplasmic YFP, or membrane CFP) specifically in club progenitor cells. The fluorescent proteins genetically tag individual cells and their descendants; therefore, progenitor cell expansion over time leads to the formation of a patch of daughter cells carrying a common fluorescent color tag (Fig. 2a). To test the effect of low-LET radiation exposure on progenitor cell dynamics, *Scgb1a1-CreER; Rosa26R-Confetti* mice were treated with tamoxifen, exposed to 1 or 5 Gy γ-rays, and the size of YFP patches was quantified. 70 days following radiation, moderate patch expansion was observed (Fig. 2b,c). Exposure to both 1 and 5 Gy γ-rays resulted in a significant increase in the number of cells per patch (Fig. 2e). Exposure to 5 Gy γ-rays resulted in a significant increase in the proportion of medium patches, containing 6–10 cells per patch (Fig. 2d). This indicates that exposure to low-LET radiation causes a subpopulation of club progenitor cells to undergo successive rounds of cell division and clonal expansion, as reflected by patch formation.

### High-LET radiation is a more potent inducer of *in vivo* airway progenitor expansion

**Figure 3:**
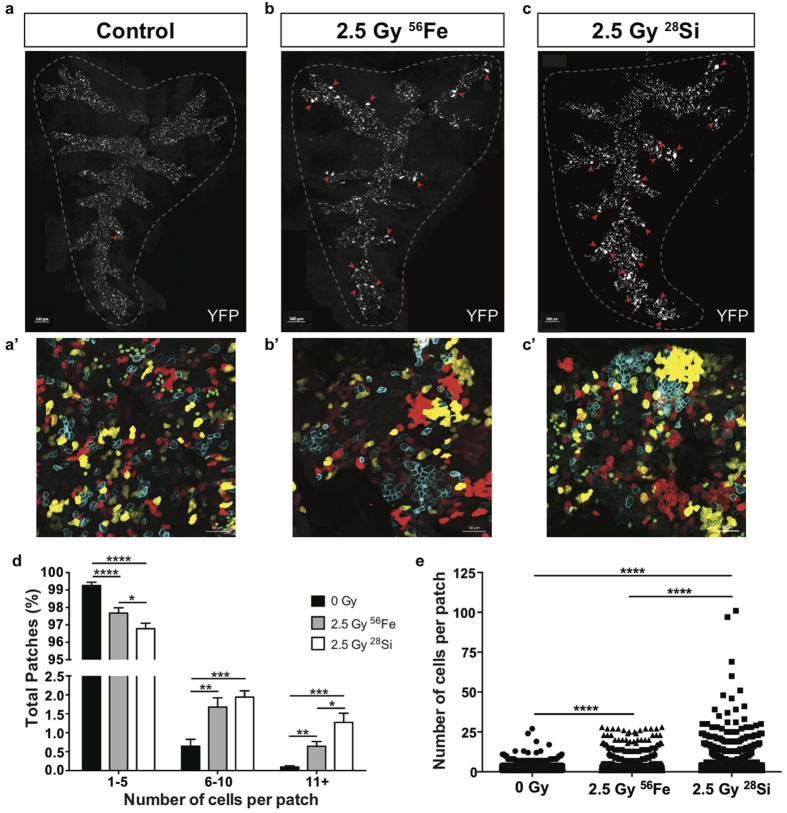
High-LET radiation induces dramatically potentiates expansion of subsets of lineage-labeled airway progenitor cells. *Scgb1a1-CreER; Rosa26R-Confetti* mice were exposed to 0 or 2.5 Gy ^56^Fe or ^28^Si ions and lungs were harvested 70 days post-radiation exposure. (**a**–**c**) Tiled image of the native YFP fluorescence (white) in a whole mount, microdissected, Sca*l*e treated accessory lobe 70 days post-^56^Fe or ^28^Si radiation exposure. Red arrows indicate medium or large patches. Scale bar represents 500 μm. (**a’**–**c’**) Whole mount 4-color fluorescence images of native confetti fluorescence (cytoplasmic RFP, nuclear GFP, cytoplasmic YFP, and membrane CFP) 70 days post-^56^Fe or ^28^Si radiation exposure corresponding to the tiled image above. Scale bar represents 50 μm. (**d**) Relative frequency of YFP patches containing various numbers of cells 70 days post-^56^Fe or ^28^Si exposure. (**e**) Number of YFP cells per patch 70 days post-^56^Fe or ^28^Si exposure. Significance of differences between groups is indicated by: *p < 0.05, **p < 0.01, ***p < 0.001, ****p < 0.0001.

The impact of radiation quality on club progenitor cell expansion was determined by exposure of *Scgb1a1-CreER; Rosa26R-Confetti* mice to high-LET radiation. We previously reported a relative biological effectiveness of 2 between X-rays and ^56^Fe ions^15^. Therefore, one week after tamoxifen treatment we exposed the *Scgb1a1-CreER; Rosa26R-Confetti* mice to 2.5 Gy of high-LET radiation to induce comparable lung injury as mice exposed to low-LET radiation. Exposure to 2.5 Gy of either ^56^Fe or ^28^Si ions resulted in a significant increase in both the proportion of medium and large patches, as well as the total patch size compared to unirradiated controls (Fig. 3a–e). Interestingly, ^28^Si ion exposure induced larger and more numerous patches than ^56^Fe ion exposure (Fig. 3d,e). To test the effect of low dose high-LET radiation exposure on progenitor cell expansion, mice were exposed to 0.2 Gy of ^56^Fe or ^28^Si ions. Although this low dose exposure increased moderate patch expansion, this difference did not reach statistical significance (Supplementary Fig. 2). Together these data suggest that high-LET radiation induces greater clonal expansion than low-LET radiation and that this differences was further impacted by ion type.

### Distal airway progenitors show enhanced sensitivity to apoptosis following high-LET radiation compared to proximal airway progenitors

**Figure 4:**
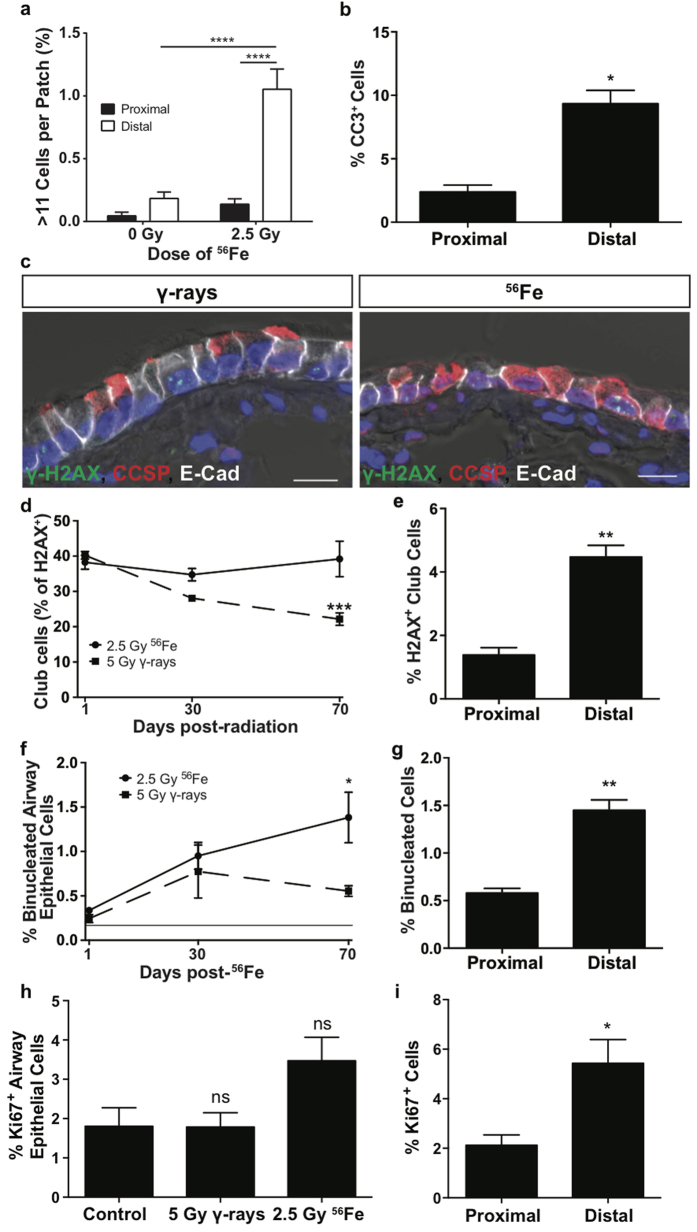
Distal airway progenitor cells undergo apoptosis, senescence, and defects in mitosis following high-LET radiation exposure. (**a**) Patches containing more than 11 cells were categorized by airway location. (**b**) The percent of cleaved-caspase 3 positive cells in the distal and proximal airway epithelium was quantitated at 1 day following 2.5 Gy ^56^Fe exposure. (**c**) Immunostaining for γ-H2AX in green, CCSP (Scgb1a1) in red, E-Cadherin in white, and DAPI in blue at 70 days post-radiation. Fluorescent channels are overlaid with a DIC image to show cilia. Scale bar represents 10 μm. (**d**) The percent of cells containing γ-H2AX foci that are club cells, as marked by CCSP (Scgb1a1), at various time points post-radiation exposure. (**e**) The percent of club cells containing γ-H2AX foci in the distal and proximal airway epithelium was quantitated at 70 days following 2.5 Gy ^56^Fe exposure. (**f**) The percent of binucleated cells, visualized using DAPI, in the airway epithelium. The solid line marks the percent of binucleated cells in the unirradiated control. (**g**) The percent of binucleated cells in the distal and proximal airway epithelium was quantitated at 70 days following 2.5 Gy ^56^Fe exposure. (**h**) The percent of proliferating cells (Ki67^+^) in the entire airway epithelium. (**i**) The percent of Ki67^+^ cells following 2.5 Gy ^56^Fe exposure in the proximal and distal airway epithelium. Significant differences between radiation type or location indicated by: ns = not significant, *p < 0.05, **<0.01, ***p < 0.001, ****p < 0.0001.

An unexpected finding from our analysis was that patch size was not uniformly impacted as a function of airway location; larger patches generated following radiation exposure were located predominately in terminal bronchioles of the distal conducting airway. We quantitated the location of large patches post-high-LET radiation exposure and found that a significant increase in the percent of large patches occurred only in the terminal bronchioles (Fig. 4a). These data suggest that clonal expansion occurs preferentially in the distal airways, indicating increased radiosensitivity in this region. We hypothesized that either progenitor cell apoptosis or senescence was driving clonal expansion and that these responses would vary by region. To determine if apoptosis was occurring post-radiation, we exposed mice to either 2.5 Gy ^56^Fe or 5 Gy γ-rays and quantified the number of airway epithelial cells positive for cleaved-caspase 3 at 1 day post-radiation. No significant increase in cleaved-caspase 3 positive cells was observed along the entire airway epithelium (Supplementary Fig. 3). However, when cleaved-caspase 3 positive cells were evaluated according to airway location, the distal terminal bronchioles contained significantly more cleaved-caspase 3 positive cells than proximal airways at 1 day post-high-LET radiation exposure (Fig. 4b).

### High-LET radiation induces persistent DNA damage in distal airway epithelial progenitor cells

To evaluate DNA damage repair, we quantified the percent of airway epithelial cells containing γ-H2AX foci at 1, 30, and 70 days post-radiation (Fig. 4c). As expected, the abundance of γ-H2AX foci increased acutely following radiation exposure and decreased over time, remaining elevated compared to baseline controls (Supplemental Fig. 4). Interestingly, there was no difference in the percent of persistent γ-H2AX foci in all epithelial cells in the airways between 5 Gy low-LET and 2.5 Gy high-LET exposures. We hypothesized that the differences in clonal expansion between radiation qualities may be due to altered repair rates of γ-H2AX foci specifically in progenitor cells. To assess this, we evaluated the proportion of club progenitor cells within γ-H2AX foci-containing cells. γ-H2AX positive club cells in mice exposed to γ-rays had an accelerated recovery rate compared to those in mice exposed to ^56^Fe (Fig. 4d). To see if club progenitor cells containing persistent γ-H2AX foci following high-LET radiation resided in a particular airway region, we evaluated γ-H2AX positive club cells according to airway location. Club cells in the terminal bronchioles contained significantly more persistent γ-H2AX foci at 70 days following high-LET radiation exposure (Fig. 4e). We next sought to determine if this response was specific to progenitor cells or if it could be observed in differentiated cells as well. To assess this, we quantified the number of persistent γ-H2AX foci following high-LET radiation exposure in post-mitotic ciliated cells. Although a greater proportion of ciliated cells contained γ-H2AX foci compared to club cells, there were no differences between airway locations (Supplemental Fig. 5). This suggests that club progenitor cells in the distal airways of mice have persistent DNA damage and may either undergo senescence or a prolonged cell cycle arrest following high-LET radiation exposure.

### Distal airway epithelial progenitor cells have defects in mitotic progression

To determine if progenitor cells that repaired their DNA damage could successfully divide, we evaluated the number of cells in the airway in mitotic arrest, as indicated by binucleated cells. The number of binucleated airway epithelial cells *in vivo* progressively increased over time following high-LET radiation, suggesting defects in mitotic progression (Fig. 4f). We categorized these binucleated cells by airway location and found that they also predominately resided in the terminal bronchioles of the distal airway at 70 days following high-LET radiation exposure (Fig. 4g). To evaluate if radiation exposure resulted in functional impairment of distal progenitor cells, we used a previously described sorting strategy to enrich for proximal and distal lung progenitor cells^16^. Sca-1^−^CD24^med^CD326^+^ distally enriched cells had a significant decrease in colony forming ability 1 day after low-LET radiation exposure when compared to Sca-1^+^CD24^med^CD326^+^ proximally enriched cells (Supplementary Fig. 6). This suggests that distal lung epithelial progenitors are more radiosensitive than their proximal airway counterparts. Together, these data show that following high-LET radiation exposure, club progenitor cells in the distal airways undergo apoptosis, senescence, and mitotic defects, which reduces the pool of progenitor cells, leading to clonal expansion.

### High-LET radiation increases proliferation in distal airway epithelial progenitor cells

We next sought to determine if the clonal expansion observed in the distal airways was associated with increased proliferation of distal airway progenitor cells. In accordance with previous results^15^, we found no significant increase in the total numbers of proliferating airway epithelial cells following low- or high-LET radiation exposure (Fig. 4h). However, when assessing proliferation according to airway location, we found that the distal airways had significantly more Ki67+ proliferating cells as compared to the proximal airways in high-LET exposed mice (Fig. 4i). Together, these results demonstrate an association between large patch formation and increased proliferation in distal airways post-high-LET radiation exposure.

### Patch expansion post-high-LET radiation is p53-dependent

**Figure 5:**
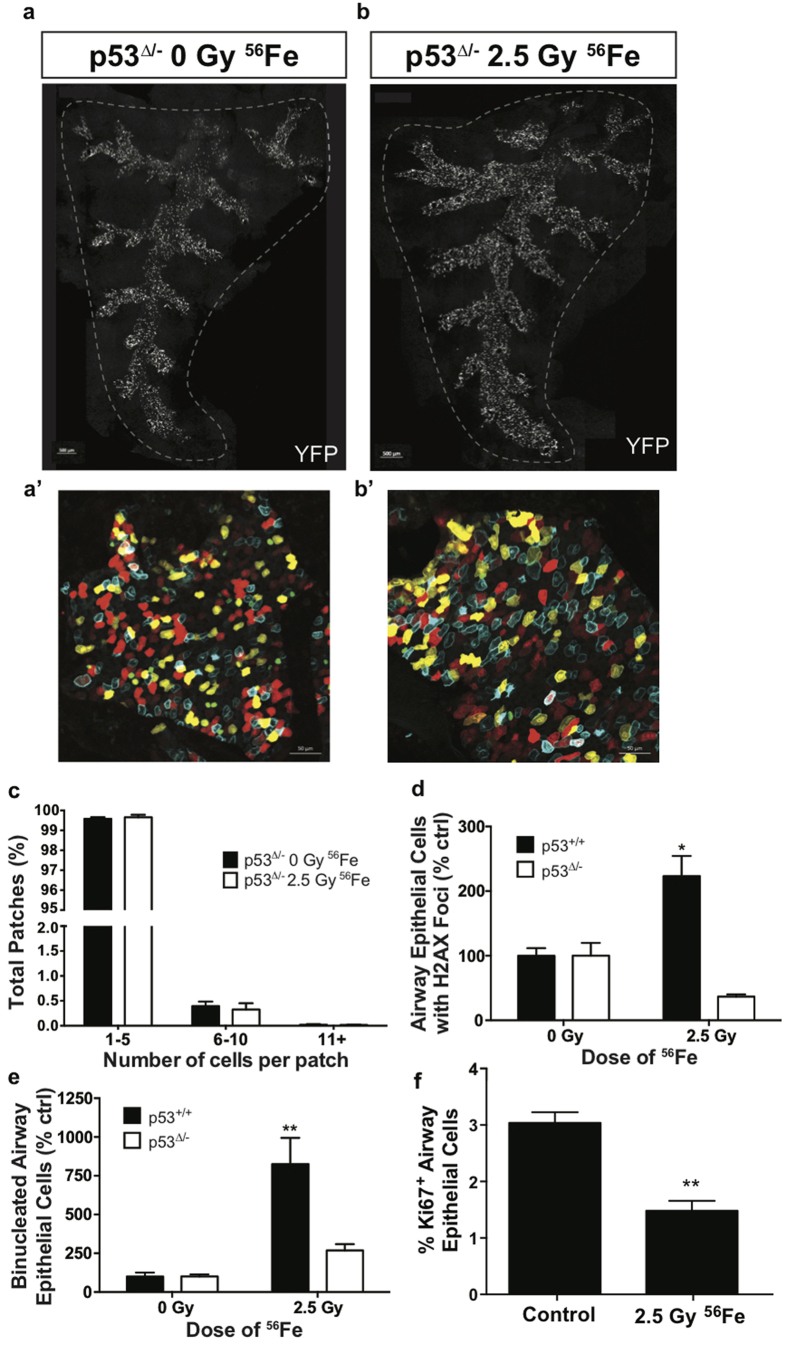
Patch expansion following high-LET radiation is p53-dependent. *Scgb1a1-CreER; Rosa26R-Confetti*, *p53*^Δ/*−*^ mice were exposed to 0 or 2.5 Gy ^56^Fe ions and tissue was collected 70 days post-radiation exposure. (**a**,**b**) Tiled image of the native YFP fluorescence (white) in a whole mount, microdissected, Sca*l*e treated accessory lobe 70 days post-^56^Fe radiation exposure. Scale bar represents 500 μm. (**a’**,**b’**) Whole mount 4-color fluorescence images of native confetti fluorescence (cytoplasmic RFP, nuclear GFP, cytoplasmic YFP, and membrane CFP) 70 days post-^56^Fe radiation exposure corresponding to the tiled image above. Scale bar represents 50 μm. (**c**) Relative frequency of YFP patches containing various numbers of cells 70 days post-^56^Fe exposure. (**d**) Percent of airway epithelial cells containing at least one H2AX foci at 70 days post-^56^Fe exposure. (**e**) The percent of binucleated cells in the airway epithelium. (**f**) The percent of proliferating (Ki67^+^) cells in the airway epithelium. p53 deficient mice showed differences in steady state compared to wild type, so some graphs are reported as a percent of the unirradiated genotypic control. Significance of differences compared to the genotypic control is indicated by: *p < 0.05; **p < 0.01.

We hypothesized that enhanced clonal expansion in subsets of club cells following exposure to high-LET ionizing radiation was the result of chronic progenitor cell deficiency and sought to determine whether p53 played role in mediating these effects. To determine if radiation-induced senescence and mitotic defects in club cells were indeed driving patch formation, we developed a conditional p53 loss-of-function mouse model. *Scgb1a1-CreER; Rosa26R-Confetti*, *p53*^ *flox−*^ mice were treated with tamoxifen to yield club cells that are deficient in p53 (p53^Δ/−^) and lineage traced by expression of one of the four fluorescent proteins from the recombined Rosa26-Confetti allele (Fig. 5a). In contrast to radiation-induced patch expansion observed in p53-sufficient mice, we observed no evidence for patch expansion 70 days post-2.5 Gy ^56^Fe exposure of mice with conditional p53 loss-of-function (Fig. 5b,c and Supplemental Fig. 7). We next assessed if p53 loss rescued the persistent DNA damage and binucleated cell phenotypes observed post-high-LET radiation exposure. Contrary to findings in p53 sufficient mice, we found no significant change in the number of γ-H2AX foci in the airway epithelium of p53^Δ/−^ mice at 70 days following ^56^Fe exposure (Fig. 5d). This was also true for appearance of binucleated cells after exposure to ^56^Fe ions, which showed no differences in abundance between p53-sufficient and -deficient mice at the 70 day recovery time point (Fig. 5e). Furthermore, the number of proliferating cells significantly decreased following exposure to ^56^Fe ions, indicating that clonal expansion is correlated with increased proliferation (Fig. 5f). These results suggest that high-LET radiation induces alterations in progenitor function that are p53-dependent.

## Discussion

High-LET radiation exposure can occur during charged particle radiotherapy, radiological accidents, and space travel. However, the risks of exposure to high-LET radiation are largely unknown. Although several studies have evaluated the effect of radiation on progenitor cell behavior using low-LET radiation^9^,^10^,^11^,^12^,^13^,^17^,^18^,^19^, very few studies have compared the effects of high-LET and low-LET radiation on stem cell behavior in any organ system. Here we show that airway epithelial progenitor cell response to radiation is quality-dependent and impacted by ion type. High-LET radiation exposure leads to a prolonged decrease in colony forming ability of club progenitor cells and a more pronounced clonal expansion than low-LET radiation. Additionally at 2.5 Gy, we show that mice exposed to 300 MeV/nucleon ^28^Si had a greater decrease in colony forming ability and more extensive clonal expansion, which is likely a reflection of increased injury, as compared to 600 MeV/nucleon ^56^Fe. This is consistent with previous studies that show ^28^Si exposure increases tumor development compared to ^56^Fe and highlights the importance of investigating radiation response in a quality-dependent manner^20^,^21^. Although the ions in the present study are not directly relevant to contemporary high-LET radiation therapy and the doses tested are higher than what would be expected on a long duration space flight to Mars, the finding that high-LET radiation induces more damage to progenitor cells than low-LET radiation should be considered when determining risk assessment from radiation exposure in both a therapeutic and occupational setting.

Many lung diseases, including radiation-induced fibrosis and tumorigenesis, involve pathological remodeling of the distal lung. This is apparent in the increased incidence of lung cancers, particularly adenocarcinomas, in atomic bomb survivors and former uranium miners exposed to high-LET particles^22^,^23^,^24^. Our data demonstrate that progenitor cells residing in distal airways are more radiosensitive than those in proximal airways, which may contribute to radiation-induced distal lung pathologies. Together our data point to a model in which high-LET radiation activates p53, leading progenitor cells in the distal airway epithelium to undergo apoptosis, senescence, and defects in mitotic progression. This decreases the pool of progenitor cells able to successfully proliferate and forces the remaining proliferative-competent progenitors to clonally expand in order to maintain homeostatic levels of turnover. One intriguing question that remains is the identity of the proliferative-competent cells. Studies in the skin, mammary gland, hematopoietic system, and intestine have revealed that resident progenitor populations have varying levels of radiosensitivity due to differential expression of p53, Bcl-2, and canonical Wnt signaling^9^,^10^,^11^,^12^,^13^,^17^,^18^,^19^. Further research is needed to determine if the club progenitors undergoing clonal expansion in the distal airway epithelium are inherently radioresistant or represent stochastically determined surviving cells.

Evidence from other organ systems suggests that cell fate decisions following radiation-induced p53 activation are tissue- and cell-type dependent^25^,^26^,^27^,^28^. Here we show that mice lacking p53 in club cells do not form patches in the airway following high-LET radiation and do not retain persistent DNA damage or show defects in mitotic progression. This finding is consistent with the role of p53 as a major regulator of cell fate; it can activate DNA repair pathways, cell cycle checkpoints through its transcriptional target p21, and can initiate apoptosis if the DNA damage is irreparable^29^,^30^,^31^. However, previous studies have shown that tissue responses to radiation can occur through p53-dependent and -independent pathways^32^,^33^. Our results suggest that airway epithelial progenitor cell response to radiation exposure is p53-dependent. Additionally, the finding that p53 loss abrogates patch formation further supports our hypothesis that clonal expansion in the distal airways results from progenitor cells undergoing apoptosis, senescence, and defects in mitotic progression.

Given that progenitor cells are thought to be the predominant cell-of-origin of cancer^6^,^34^, clonal expansion likely describes an early event along the path to radiation-induced carcinogenesis. Previous studies have implicated clonal expansion as an important step in carcinogenesis^35^,^36^,^37^,^38^. One hypothesis about the mechanism of how this occurs is that clonal expansion of a progenitor cell carrying a mutation in an oncogene or tumor suppressor would lead to the generation of a field of cells all carrying the same mutation. This field effect might lead to further mutations and eventually oncogenic transformation. However, precisely how this leads to tumor development is still unknown.

In conclusion, this work reveals that distal airway epithelial cells have prolonged functional impairments and altered clonal dynamics following high-, but not low-LET radiation exposure. This knowledge deepens our understanding of the extent of normal tissue damage following various qualities of radiation, which has implications for high-LET radiation therapy and for more accurately predicting the risk to patients and astronauts exposed to high energy and charge (HZE) radiation.

## Methods

### Mice

The *Scgb1a1-CreER; Rosa26R-Confetti* mice were established by crossing *Scgb1a1-CreER* mice (kindly provided by Brigid L.M. Hogan, Duke University) with *Rosa26R-Confetti* mice (JAX stock number 017492) as previously reported^16^. These mice were crossed to p53^flox^ (JAX stock number 008462) and p53^−/−^ (JAX stock number 002101) mice to generate *Scgb1a1-CreER*; *Rosa26R-Confetti*, *p53*^ *flox−*^ mice. Confetti mice were injected i.p. 3 times every other day with 200 mg/kg body weight tamoxifen in corn oil to randomly introduce one of four genetic tags into the *Scgb1a1*-expressing progenitor cells and recombine the p53 allele to generate progenitor cells deficient for p53. *In vitro* experiments used mice heterozygous for *Rosa26R-mTmG* (JAX stock number 007576). All mice were maintained and treatments were carried out according to Cedars-Sinai Medical Center IACUC approved protocols. All methods used in this manuscript were approved by Cedars-Sinai Medical Center IACUC.

### Radiation exposure

Mice, eight to twelve weeks old, were either exposed to γ-rays, ^56^Fe, or ^28^Si radiation. For low-LET irradiation, unanesthetized mice were exposed whole body to 1 or 5 Gy of γ-rays (Gammacell 40 Exactor, dose rate 1 Gy/min). For high-LET irradiation, mice were exposed whole body to 0.2, 0.5, 1, or 2.5 Gy of 600 MeV/nucleon ^56^Fe ions or 300 MeV/nucleon ^28^Si ions (NASA Space Research Laboratory’s linear accelerator at Brookhaven National Laboratory, dose rate 0.2 Gy/min).

### *In vitro* cultures

Airway epithelial cell isolation and flow cytometry was performed as previously described by Farin *et al*.^15^. Briefly, 5,000 sorted EpCAM^+^,CD31/34/45^−^,7AAD^−^ epithelial cells were mixed with 100,000 unirradiated immortalized mouse fibroblasts. The mixture was added to an equal volume of growth factor reduced Matrigel (BD Biosciences) and seeded to the apical surface of 24-well transwell filter inserts (BD Biosciences) placed in 24-well flat-bottom culture plates. The solution was allowed to polymerize for 30 min at 37 °C, then basic medium was added to the basal compartment of the well. Cell cultures were maintained for 14 days at 37 °C in a humidified incubator (5% CO_2_). Colony-forming efficiency was calculated as the percentage of seeded cells that give rise to colonies, imaged on a Zeiss Axiovert40 fluorescent microscope and quantitated using FIJI. Enrichment for CD24^med^, Sca-1^−^ distal and CD24^med^, Sca-1^+^ proximal airway epithelial cells was performed as previous described^16^.

### Whole mount imaging and quantification

All images were taken using a Zeiss 780 confocal microscope. For patch expansion experiments, accessory lobes from at least 3 independent animals were treated with Scale to clarify tissue^39^. The lobes were microdissected to expose the airways and the native fluorescent proteins were imaged. Patch size of YFP+ patches was quantified using Imaris (Bitplane), with the number of contiguous YFP+ cells defining a patch. The quantitation presented in Figs 2, 3 and 5 are based on only the YFP patches and not the three other fluorescent proteins.

### Immunofluorescence staining and quantification

Five-micrometer sections were collected from lung tissue fixed with 10% NBF. De-waxing, antigen retrieval, and blocking was performed and the sections were incubated with primary antibodies at 4 °C overnight. The following antibodies were used: Mouse IgG1 anti-phospho-Histone H2AX (Ser139) (1:500, Milipore), Rabbit anti-CCSP (1:10,000, in house), Mouse IgG2a anti-E-Cadherin (1:1000, BD Biosciences), Rabbit anti-Cleaved Caspase-3 (Asp175) (1:500, Cell Signaling Technology), Rabbit anti-Ki67 (1:500, Abcam). Sections were washed with PBS and incubated with fluorochrome-conjugated secondary antibody and DAPI for 30 minutes at room temperature. Sections were washed again and mounted in Fluormount G. Sections of tissue from 3–5 independent animals were imaged and between 500 and 1000 cells per animal was quantitated using FIJI. For proximal and distal analysis, distal airways were categorized as 100 cells from a bronchioalveolar duct junction and proximal airways were categorized as the cells along a primary airway branch.

### Statistical analysis

Data were analyzed and compared between groups using a one-way ANOVA or two-way ANOVA with post-hoc analysis (Prism, GraphPad). p < 0.05 was considered statistically significant and is presented as *p < 0.05, **p < 0.01, ***p < 0.001 or ****p < 0.0001.

## Additional Information

**How to cite this article**: McConnell, A. M. *et al*. Distal airway epithelial progenitor cells are radiosensitive to high-LET radiation. *Sci. Rep.* **6**, 33455; doi: 10.1038/srep33455 (2016).

## Supplementary Material

Supplementary Information
