# Supplementary material for: Distal airway epithelial progenitor cells are radiosensitive to High-LET radiation
Source: Sci Rep. 2016 Sep 23;6:33455. doi: 10.1038/srep33455 (PMC5034250; doi:10.1038/srep33455)

Supplemental Information

Distal airway epithelial progenitor cells are radiosensitive to high-LET radiation.

Alicia M. McConnell[1,2], Bindu Konda[2], David G. Kirsch[3], and Barry R. Stripp[1,2]

Supplementary Figure Legends

Supplementary Figure 1. ^{28}Si ions reduce colony forming ability more than ^{56}Fe ions. Mice were exposed to 0, 0.5, 1, or 2.5 Gy ^{56}Fe or ^{28}Si ions and epithelial cells were isolated 1 day post-radiation exposure. Colony forming efficiency of epithelial cells isolated from mice at various time points following radiation exposure. Colony forming efficiency is expressed as a percent of the unirradiated control. Significant differences between radiation type is indicated by: *$p < 0.05$; **$p < 0.01$.

Supplementary Figure 2. No significant increase in patch expansion after low dose high-LET radiation exposure. *Scgb1a1-CreER; Rosa26R-Confetti* mice were exposed to 0 or 0.2 Gy ^{56}Fe or ^{28}Si ions and tissue was collected 70 days post-radiation exposure. Relative frequency of YFP patches containing various numbers of cells 70 days post-^{56}Fe or ^{28}Si exposure.

Supplementary Figure 3. No significant increase in apoptosis is observed in the airway epithelium following radiation exposure. Mice were exposed to 2.5, or 5 Gy high- or low-LET radiation respectively and tissue was collected 1 day post-radiation exposure and stained for cleaved-caspase 3. ns = not significant.

Supplementary Figure 4. No significant difference in DNA damage following low- or high-LET radiation exposure. Mice were exposed to 2.5, or 5 Gy high- or low-LET radiation and sacrificed at 1, 30, or 70 days. No difference in the number of cells containing γ-H2AX foci was observed. Graphed as a percent of control, as indicated by the solid line.

Supplementary Figure 5. No significant difference in persistent DNA damage in ciliated cells between airway locations. Mice were exposed to 2.5 Gy ^{56}Fe and sacrificed at 70 days. No difference in the number of ciliated cells with persistent γ-H2AX foci residing in proximal or distal airways was observed. ns = not significant.

Supplementary Figure 6. Distally enriched progenitors are more radiosensitive than proximally enriched progenitors. Mice were exposed to various doses of low-LET radiation and CD24^{med}, Sca-1^{+} proximally enriched cells or CD24^{med}, Sca-1^{-} distally enriched cells were isolated 1 day post-radiation exposure. Colony forming efficiency was evaluated at day 14 *in*

vitro. Colony forming efficiency is expressed as a percent of the unirradiated control. Significance to unirradiated control is indicated by: *$p < 0.05$, ****$p < 0.0001$.

Supplementary Figure 7. No significant change in patch size occurs in high-LET exposed p53-deficient mice. *Scgb1a1-CreER; Rosa26R-Confetti,p53$^{\Delta/-}$* mice were exposed to 0 or 2.5 Gy ^{56}Fe ions and tissue was collected 70 days post-radiation exposure. Number of YFP cells per patch 70 days post-^{56}Fe exposure. ns = not significant.

Supplemental Figure 1

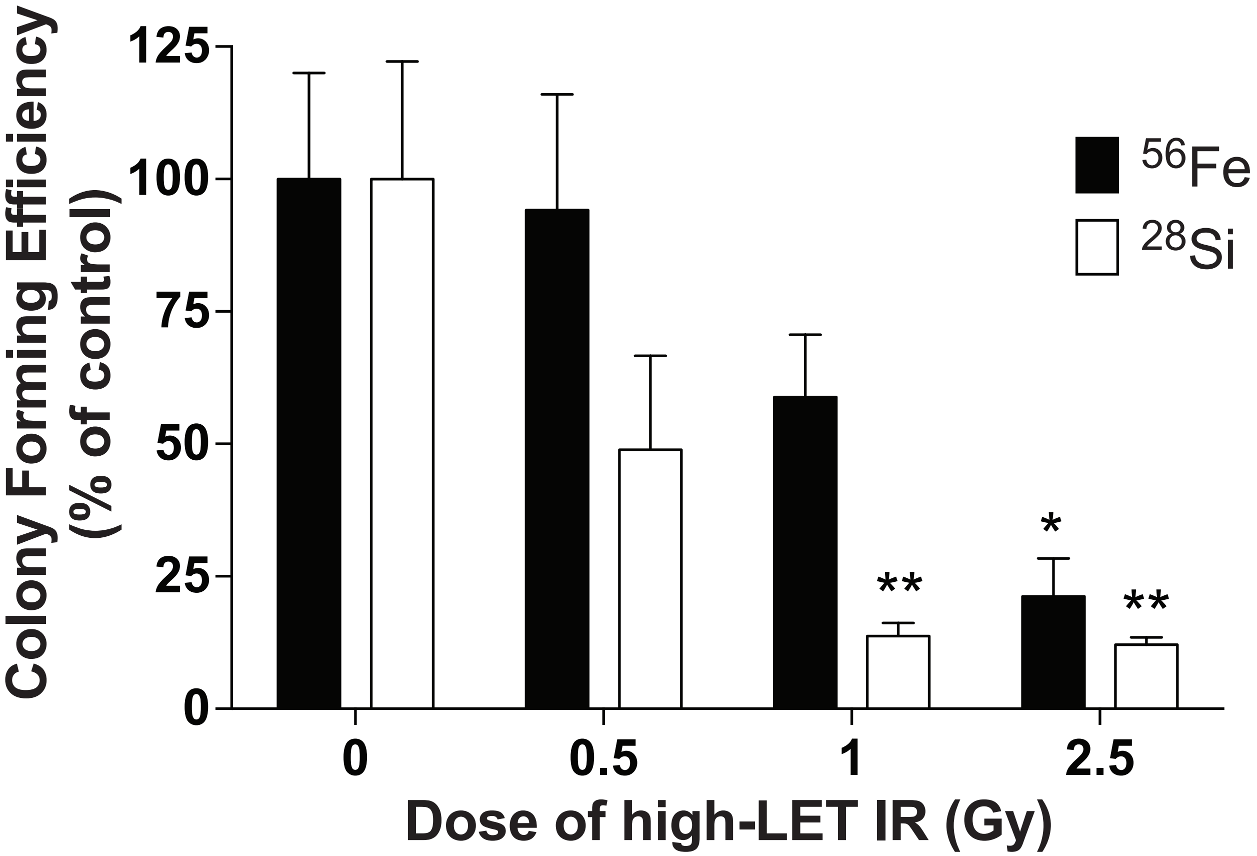

Supplemental Figure 2

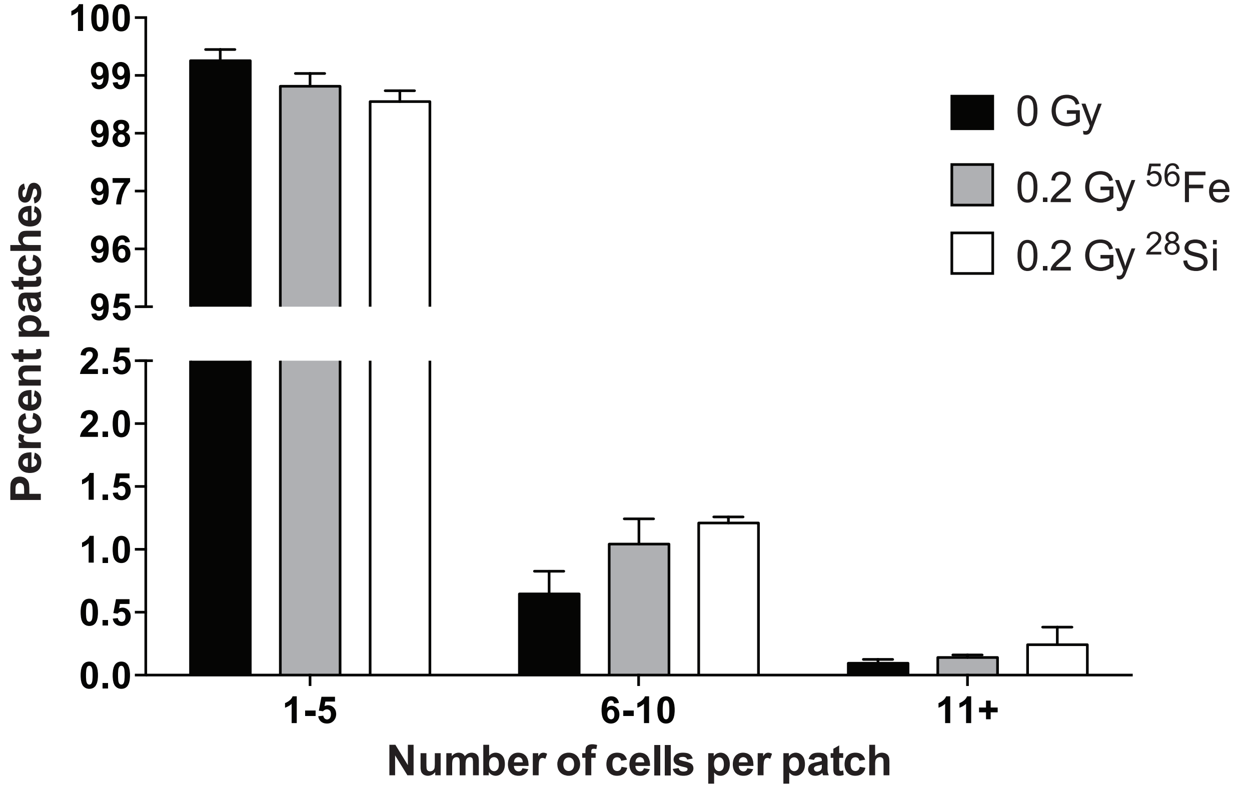

Supplemental Figure 3

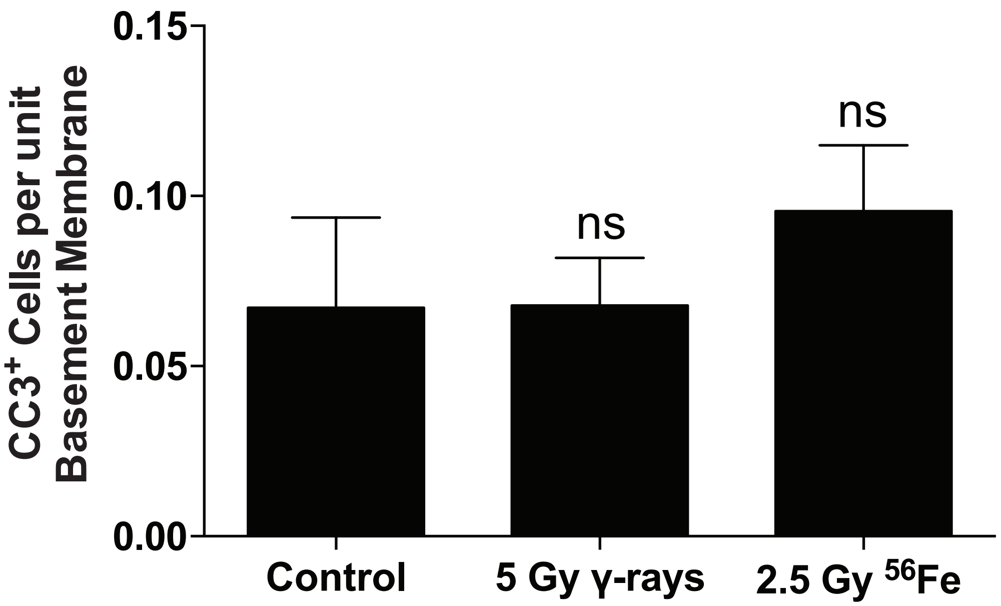

Supplemental Figure 4

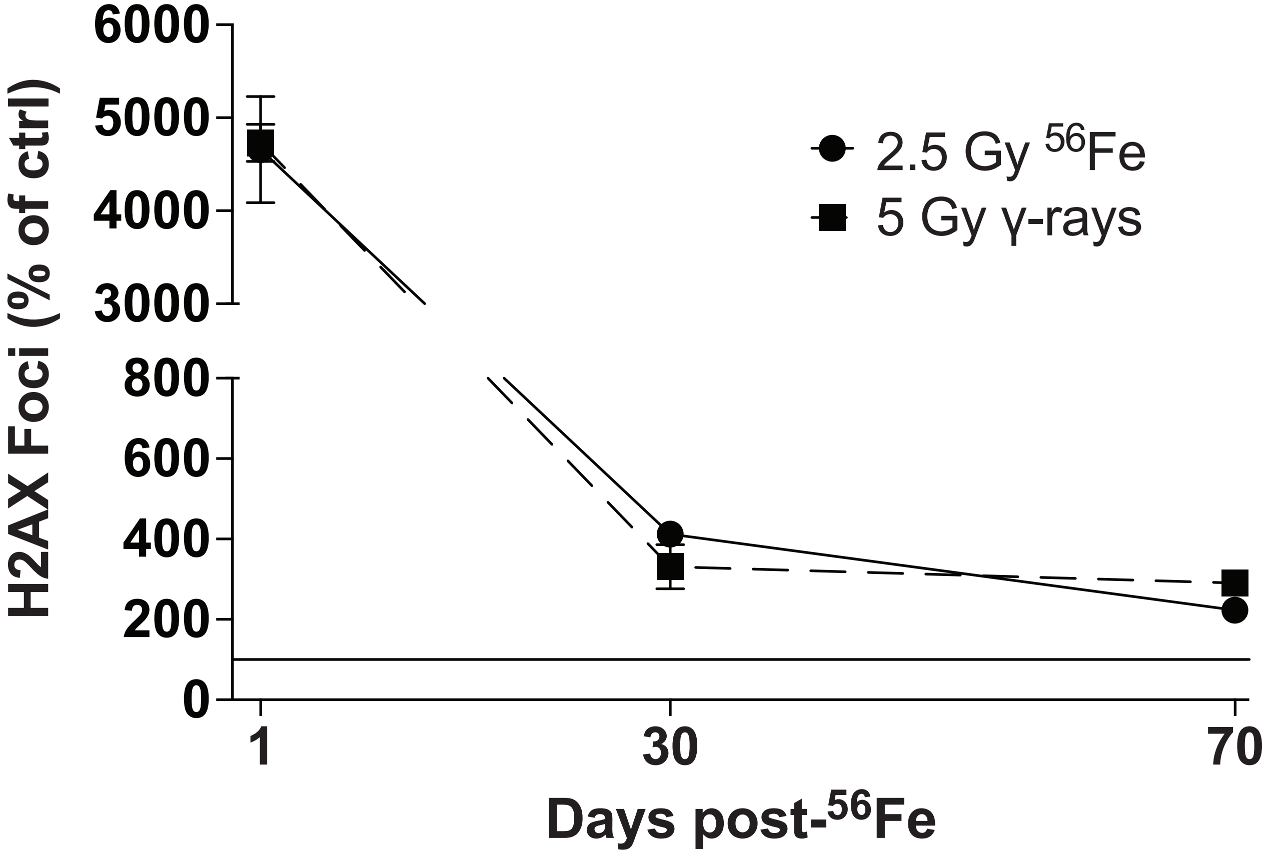

Supplemental Figure 5

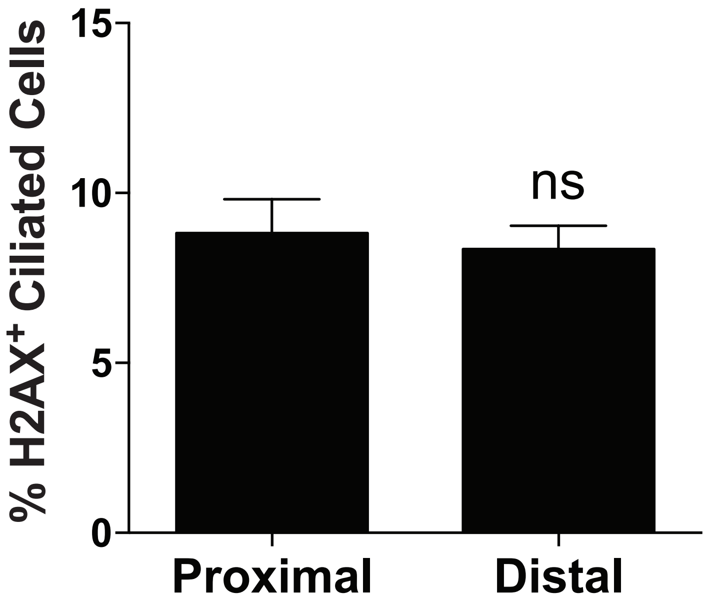

Supplemental Figure 6

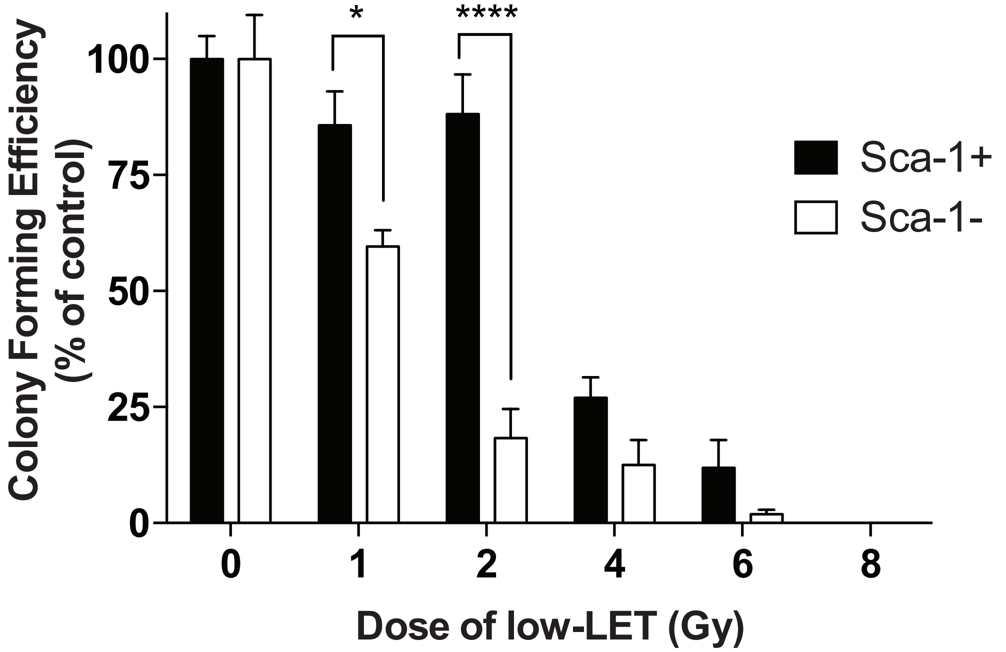

Supplemental Figure 7

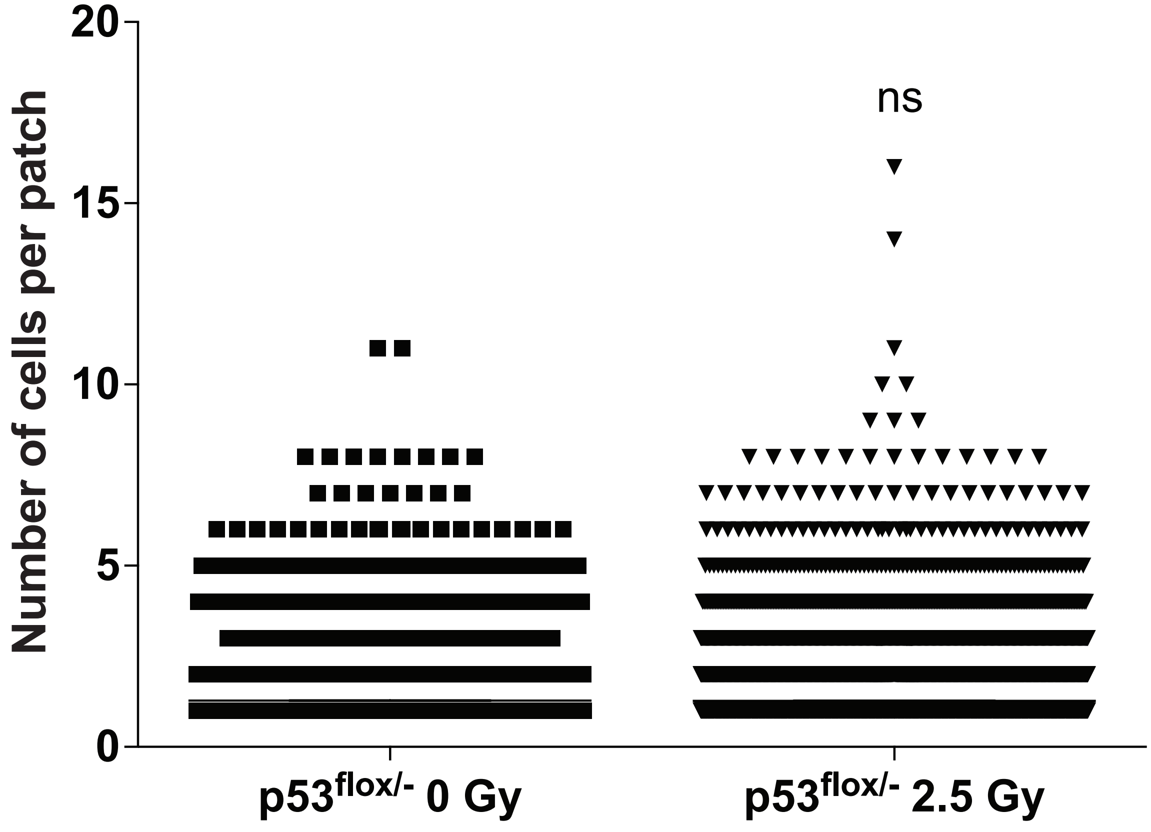

Supplement: Supplementary Information [file srep33455-s1.pdf]
